# Correlation between immune-related adverse events and prognosis in patients with gastric cancer treated with nivolumab

**DOI:** 10.1186/s12885-019-6150-y

**Published:** 2019-10-21

**Authors:** Ken Masuda, Hirokazu Shoji, Kengo Nagashima, Shun Yamamoto, Masashi Ishikawa, Hiroshi Imazeki, Masahiko Aoki, Takahiro Miyamoto, Hidekazu Hirano, Yoshitaka Honma, Satoru Iwasa, Natsuko Okita, Atsuo Takashima, Ken Kato, Narikazu Boku

**Affiliations:** 1 Gastrointestinal Medical Oncology Division, National Cancer Center Hospital, 5-1-1 Tsukiji, Chuo-ku, Tokyo, 104-0045 Japan; 2 Research Center for Medical and Health Data Science, The Institute of Statistical Mathematics, Tokyo, Japan; 3 Keio University School of Medicine, Tokyo, Japan

**Keywords:** Gastric cancer, Immune-related adverse events, Nivolumab, Programmed cell death-1

## Abstract

**Background:**

Recent studies have shown that immune-related adverse events (irAEs) caused by immune checkpoint inhibitors were associated with clinical benefit in patients with melanoma or lung cancer. In advanced gastric cancer (AGC) patients, there have been few reports about the correlation between irAEs and efficacy of immune checkpoint inhibitors. In this study, we retrospectively investigated the correlation between irAEs and efficacy in AGC patients treated with nivolumab.

**Methods:**

The subjects of this study were AGC patients received nivolumab monotherapy between January 2015 and August 2018. IrAEs were defined as those AEs having a potential immunological basis that required close follow-up, or immunosuppressive therapy and/or endocrine therapy. We divided the patients who received nivolumab into two groups based on occurrence of irAEs; those with irAEs (irAE group) or those without (non-irAE group). We assessed the efficacy in both groups**.**

**Results:**

Of the 65 AGC patients that received nivolumab monotherapy, 14 developed irAEs. The median time to onset of irAEs was 30.5 days (range 3–407 days). Median follow-up period for survivors was 32 months (95% CI, 10.8 to 34.5). The median progression-free survival was 7.5 months (95% CI, 3.6 to 11.5) in the irAE group and 1.4 months (95% CI, 1.2 to 1.6) in the non-irAE group (HR = 0.11, *p* < 0.001). The median overall survival was 16.8 months (95% CI, 4.4 to not reached) in the irAE group and 3.2 months (95% CI, 2.2 to 4.1) in the non-irAE group (HR = 0.17, *p* < 0.001). Multivariate analysis demonstrated that number of metastatic sites ≥2 (HR = 2.15; 95% CI, 1.02 to 4.54), high ALP level (HR = 2.50; 95% CI, 1.27 to 4.54), and absence of irAEs (HR = 9.54, 95% CI, 3.34 to 27.30 for yes vs. no) were associated with a poor prognosis. The most frequent irAEs was diarrhea/colitis (*n* = 5). Grade 3 adverse events were observed in 6 patients; hyperglycemia (*n* = 2), diarrhea/colitis (*n* = 1), adrenal insufficiency (*n* = 1), aspartate aminotransferase increased (*n* = 1), peripheral motor neuropathy (*n* = 1). There were no grade 4 or 5 adverse events related to nivolumab.

**Conclusions:**

Development of irAEs was associated with clinical benefit for AGC patients receiving nivolumab monotherapy.

## Background

While the mortality rate of gastric cancer has been continuously decreasing, it remains one of leading causes of cancer deaths worldwide and was reported to be especially high in East Asia [1, 2]. In Japan, gastric cancer is the most common malignant disease in men and the third ranking cancer in terms of incidence in women, while also exhibiting the second highest mortality rate. For unresectable or recurrent advanced gastric cancer (AGC), systemic chemotherapy is of crucial importance in order to obtain palliation of symptoms and improvement in survival. However, the prognosis for patients with AGC remains poor with median survival times of 10–13 months [3, 4].

Nivolumab, a monoclonal antibody targeting programmed cell death-1 (PD-1), has been shown to provide remarkable efficacy for patients with various malignant tumors [5–11]. Nivolumab has been recently recognized as a standard of care in several carcinomas. Regarding gastric cancer, the ATTRACTION-2 study was carried out in order to investigate the efficacy and safety of nivolumab for heavily pretreated patients with AGC [12]. This randomized, double-blind and placebo-controlled phase 3 trial showed superiority of nivolumab over placebo, associated with an objective response rate (ORR) of 11.2% (95% CI, 7.7 to 15.6), median progression-free survival (PFS) of 1.61 months (95% CI, 1.54 to 2.30) and median overall survival (OS) of 5.26 months (95% CI 4.60 to 6.37). Based on the results of this study, nivolumab was approved for AGC as third- or later line treatment in Japan.

Immune checkpoint inhibitors such as nivolumab cause imbalances in immunological tolerance, resulting in inflammatory side effects which are called immune-related adverse events (irAEs) [13, 14]. IrAEs are dissimilar from AEs experienced with conventional systemic chemotherapy. In previous studies, irAEs have been defined as AEs with a potential immunologic cause and with necessity of frequent monitoring, or immunosuppressive and/or endocrine therapy according to the severity of the respective AE [6, 14–16]. Recently, several studies have shown that irAEs were associated with efficacy of anti-PD-1 antibody treatment in patients with melanoma and non–small cell lung cancer [17–24].

In contrast, few data are available on this relationship in AGC patients. Therefore, in this study, we retrospectively investigated the correlation between irAEs and efficacy in AGC patients treated with nivolumab.

## Methods

### Patients

AGC patients with histologically confirmed adenocarcinoma who were treated with nivolumab monotherapy between January 2015 and August 2018 at National Cancer Center Hospital were identified from the database, and patients who received previous treatment with immunotherapy were excluded. We reviewed the medical records and the following characteristics of patients were collected: age, gender, Eastern Cooperative Oncology Group performance status (ECOG PS), histology, history of gastrectomy, metastatic sites, presence of target lesion according to the response evaluation criteria in solid tumors (RECIST) version 1.1, baseline blood cell count and serum alkaline phosphatase (ALP) level [25] before initiating nivolumab treatment. The neutrophil-to-lymphocyte ratio (NLR) was calculated by dividing the lymphocyte count into neutrophil count. IrAEs were defined as mentioned above. We divided the patients treated with nivolumab into two groups based on occurrence of irAEs; those with irAEs (irAE group) or those without (non-irAE group). We compared the efficacy between the irAE and non-irAE groups.

The study protocol was reviewed and approved by the institutional ethics committee of the National Cancer Center Hospital. Due to the retrospective nature of this study, informed consent was not obtained from each patient.

### Treatment and assessment

Patients received the standard nivolumab dose of 3 mg/kg intravenously every 2 weeks until disease progression, clinical deterioration, unacceptable toxicity, or patient’s refusal. In relation to safety analysis, we evaluated adverse events linked to nivolumab use according to National Cancer Institute Common Terminology Criteria for Adverse Events ver. 4.03. Objective tumor response was evaluated in patients who had target lesions according to the RECIST version 1.1, with assessment by computed tomography scan repeated every 6 to 8 weeks after nivolumab therapy.

### Statistical analysis

Differences between the two groups were compared using the Fisher’s exact tests for categorical variables. PFS was defined as the time from the beginning of nivolumab treatment to progression or death from any cause; PFS was censored at the date verifiable to be progression free, and patients whose treatment discontinued due to toxicity without disease progression were censored at the beginning of the next treatment including best supportive care. OS was measured until death or censored at the latest follow-up for surviving patients. Probabilities of survival were estimated using the Kaplan–Meier method and compared using the log-rank test. In addition, landmark analysis at 2 months after initiating nivolumab was performed to adjust effects of early progression or death, in which patients who had events up to 2 months were excluded. Univariate analysis and multivariate analysis using a Cox proportional hazards regression model were performed to explore prognostic factors for survival; the change-in-estimate (CIE) method [26] was used to assess the influence of prognostic factors. All statistical analyses were performed using JMP version 14.0 (SAS Institute, Cary, NC, USA) and SAS version 9.4 (SAS Institute Incorporated, Cary, NC, USA). All *P* values are two-sided, and *P* < 0.05 was considered to indicate a statistically significant difference.

## Results

### Patient characteristics

Sixty-nine patients with AGC who were treated with nivolumab were identified to act as the source of the subjects to be used in this study. Among them, 65 patients were selected in our study. Four patients were excluded because of their histologic types: squamous cell carcinoma (*n* = 1) and neuroendocrine carcinoma (*n* = 3). The median patient age was 66 years (range, 35–83), and 59 patients (90.8%) had an ECOG PS of 0 or 1. The median ALP level was 342 (range, 182–3013).

### Clinical course of all patients

**Fig. 1:**
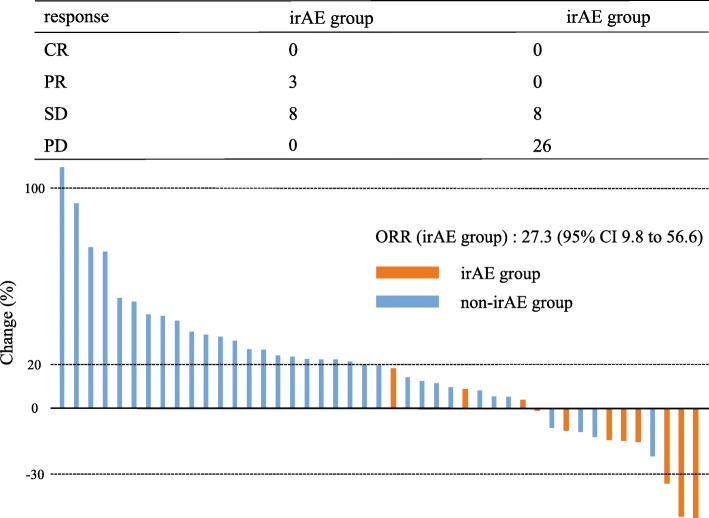
Responses to nivolumab based on maximal percentage of tumor reduction (*N* = 45)

Median follow-up period for survivors was 32 months (95% CI, 10.8 to 34.5). Fifty-four (83.1%) of the 65 patients died. The median survival time (MST) was 4.0 months (95% CI 3.1 to 5.5), and the median PFS was 1.6 months (95% CI 1.4 to 2.8). Among 45 patients who had target lesions, partial response (PR) was achieved in 3 patients and stable disease (SD) was observed in 16 patients, resulting in an ORR of 6.7% (95% CI, 2.3 to 17.9) and disease control rate of 42.3% (95% CI, 29.0 to 56.7). Figure 1 shows a waterfall plot indicating the best responses to nivolumab.

### Comparison between irAE and non-irAE groups

The patient background of the irAE and non-irAE groups are summarized in Table 1. No significant differences in clinical profiles, apart from ECOG PS, were observed between the two groups. White blood cell and neutrophil count at baseline in the irAE group tended to be low compared to that in the non-irAE group, but there was no significant difference between the two groups.

**Table 1:** Characteristics of patients in irAE and non-irAE groups

	All patients No. (%)	irAE group No. (%)	non-irAE group No. (%)	*P*-value
Total N	65	14	51	
Age				
< 65	28 (43.1)	4 (28.6)	24 (47.1)	0.24
≥ 65	37 (56.9)	10 (71.4)	27 (52.9)	
Sex				
Female	14 (21.5)	6 (42.9)	8 (15.7)	0.06
Male	51 (78.5)	8 (57.1)	43 (84.3)	
ECOG PS				
0	7 (10.8)	4 (28.6)	3 (5.9)	0.03
≥ 1	58 (89.2)	10 (71.4)	48 (94.1)	
Number of metastatic sites				
< 2	16 (24.6)	3 (21.4)	13 (25.5)	1.00
≥ 2	49 (75.4)	11 (78.6)	38 (74.5)	
ALP				
Low	31 (47.7)	7 (50.0)	24 (47.1)	1.00
High	34 (52.3)	7 (50.0)	27 (52.9)	
Histologic type				
Intestinal	34 (52.3)	9 (64.3)	25 (49.0)	0.37
Diffuse	31 (47.7)	5 (35.7)	26 (51.0)	
HER2 status				
Positive	13 (20.0)	11 (78.6)	41 (80.4)	1.00
Negative	52 (80.0)	3 (21.4)	10 (19.6)	
Disease status				
Stage IV	32 (49.2)	4 (28.6)	28 (54.9)	0.13
Recurrence	33 (50.8)	10 (71.4)	23 (45.1)	
NLR				
Low (< 4)	37 (56.9)	8 (57.1)	29 (56.9)	0.96
High (≥4)	28 (43.1)	6 (42.9)	22 (43.1)	
Baseline blood cell count median (range)				
WBC (/μL)	3900 (2500–19,900)	4900 (3700–14,300)	6300 (2500–19,900)	0.06
Neutrophil (/μL)	2570 (1310–18,710)	3210 (2180–9880)	4290 (1310–18,710)	0.06
Lymphocyte (/μL)	965 (400–3230)	1080 (650–2820)	1080 (650–3230)	0.67
Eosinophil (/μL)	91 (0–839)	121 (18–684)	91 (0–839)	0.22
NLR	2.60 (1.00–31.2)	2.92 (1.00–6.33)	3.54 (1.16–31.2)	0.21

*ALP* alkaline phosphatase, *ECOG PS* Eastern Cooperative Oncology Group Performance Status, *irAE* immune-related adverse event, *NLR* neutrophil-to-lymphocyte ratio, *WBC* white blood cell

**Fig. 2:**
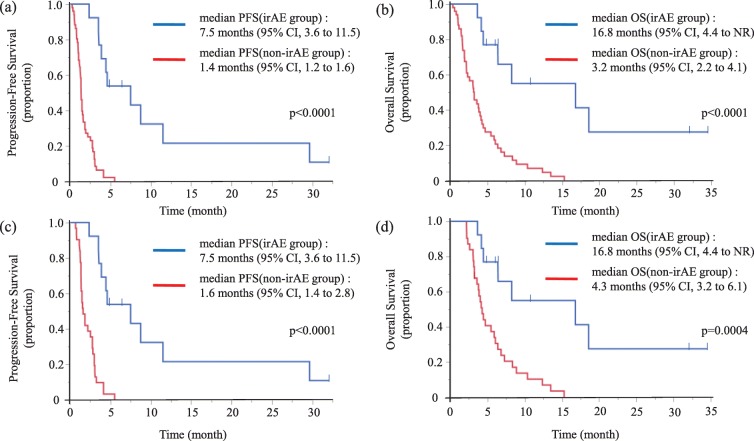
Kaplan-Meier survival curve of progression-free survival (PFS) and overall survival (OS). PFS (**a**) and OS (**b**) following nivolumab treatment in non-irAE group (*N* = 51) and irAE group (*N* = 14); PFS (**c**) and OS (**d**) following nivolumab treatment in non-irAE group (*N* = 31) and irAE group (*N* = 14) by landmark time (2 months)

In the irAE group, the best overall responses were PR in 3 patients and SD in 8 patients, resulting in an ORR of 27.3% (95% CI, 9.8 to 56.6). The Kaplan-Meier curves of PFS and OS in the irAE and the non-irAE groups are shown in Fig. 2. Median PFS was 7.5 months (95% CI, 3.6 to 11.5) in the irAE group and 1.4 months (95% CI, 1.2 to 1.6) in the non-irAE group [hazard ratio (HR) = 0.11, *p* < 0.001], respectively. The median OS was 16.8 months (95% CI, 4.4 to not reached) in the irAE group and 3.2 months (95% CI, 2.2 to 4.1) in the non-irAE group (HR = 0.17, *p* < 0.001). In addition, we performed a landmark analysis which evaluated the PFS and OS by excluding patients who had events (death) within 2 months (Fig. 2). Even in this subgroup, the PFS and OS were significantly longer in patients experiencing irAEs. After excluding the patients who had events within one and 3 months, similar results were observed showing that the irAE group had longer OS and PFS than the non-irAE group (data not shown).

In the univariate analysis with age (≥65 or < 65), gender (male or female), PS (≥1 or < 1), the number of metastases (≥2 or < 2), ALP level (high or normal), histologic type (diffuse or intestinal), HER2 (positive or negative), disease status (stage 4 or recurrence) and occurrence of irAEs (non-irAE group or irAE group) as covariates, ALP high and non-irAE group were significantly associated with shorter OS. Multivariate analysis demonstrated that number of metastatic sites ≥2 (HR = 2.15; 95% CI, 1.02 to 4.54), high ALP level (HR = 2.50, 95% CI, 1.27 to 4.54), and absence of irAEs (HR = 9.54, 95% CI, 3.34 to 27.30) were associated with a poor prognosis (Table 2).

**Table 2:** Univariate and multivariate analyses of OS with Cox regression models

Covariate	Univariate analysis (*n* = 65)				Multivariate analysis (*n* = 65)			
	HR	95% C.I.		*P*-value	HR	95% C.I.		*P*-value
Group								
irAE	Reference				Reference			
non-irAE	6.081	2.373	15.582	< 0.001	9.543	3.336	27.302	< 0.001
ECOG PS								
0	Reference				Reference			
≥ 1	2.673	0.960	7.444	0.060	1.271	0.425	3.805	0.622
Number of metastatic sites								
< 2	Reference				Reference			
≥ 2	1.465	0.763	2.812	0.251	2.147	1.016	4.538	0.045
ALP								
Low	Reference				Reference			
High	2.259	1.284	3.97.3	0.005	2.499	1.272	4.913	0.008
Disease status								
Recurrence	Reference				Reference			
Stage IV	1.584	0.915	2.743	0.101	0.813	0.420	1.577	0.541
NLR								
Low (< 4)	Reference				Reference			
High (≥4)	1.716	0.991	2.971	0.054	1.551	0.810	2.971	0.185

*ALP* alkaline phosphatase, *ECOG PS* Eastern Cooperative Oncology Group Performance Status, *irAE* immune-related adverse event

### Toxicity

**Fig. 3:**
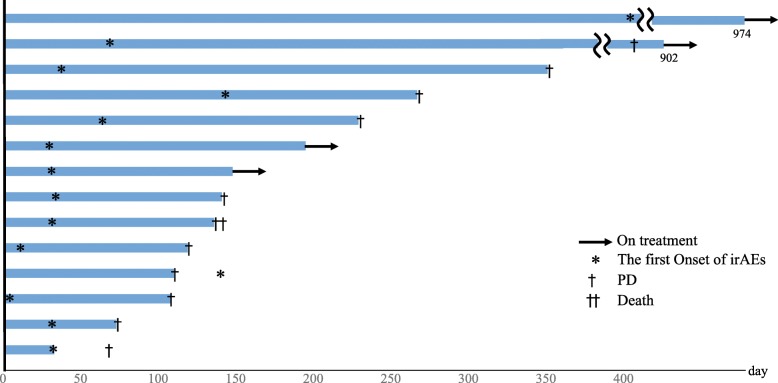
Swimmer’s plot of the duration of treatment with nivolumab in irAE group (*N* = 14)

Fourteen of the 65 patients (21.5%) experienced irAEs in our study. Details of these irAEs are shown in Table 3. The most frequent adverse event was diarrhea/colitis (*n* = 5). Grade 3 adverse events were observed in 6 patients; hyperglycemia (*n* = 2), diarrhea/colitis (*n* = 1), adrenal insufficiency (*n* = 1), aspartate aminotransferase increased (*n* = 1), peripheral motor neuropathy (*n* = 1). The median time to onset of irAEs was 30.5 days (range 3–407 days). One of the 14 patients experienced the irAE after discontinuation of nivolumab due to progression of disease. There were no grade 4 or 5 adverse events related to nivolumab. Table 4 shows details of the patients who experienced irAEs (*n* = 14) and clinical outcomes after immunosuppressive therapies or endocrine therapies. Figure 3 summarizes the duration of the treatment with nivolumab observed in the irAE group. One patient with grade 3 pneumonitis discontinued nivolumab while the others continued nivolumab after occurrence of irAEs.

**Table 3:** Categorization of irAEs

irAEs	No. (%)	Median days to onset	Grade of irAEs, n, 1/2/3/4
Diarrhea/colitis	5 (7.7)	60.0	2/2/1/0
Hyperglycemia	2 (3.1)	398.5	0/0/2/0
Pruritus	2 (3.1)	50.0	2/0/0/0
Rash	2 (3.1)	12.0	1/1/0/0
Type 1 DM	2 (3.1)	398.5	0/2/0/0
Adrenal insufficiency	1 (1.5)	143.0	0/0/1/0
ALT increased	1 (1.5)	28.0	0/1/0/0
AST increased	1 (1.5)	28.0	0/0/1/0
Appetite loss	1 (1.5)	158.0	1/0/0/0
Hypothyroidism	1 (1.5)	167.0	1/0/0/0
Dry skin	1 (1.5)	29.0	1/0/0/0
Edema limbs	1 (1.5)	28.0	1/0/0/0
Myalgia	1 (1.5)	16.0	0/1/0/0
Peripheral motor neuropathy	1 (1.5)	3.0	0/0/1/0
Pneumonitis	1 (1.5)	32.0	0/1/0/0
QTc interval prolonged	1 (1.5)	42.0	1/0/0/0

*ALT* alanine aminotransferase, *AST* aspartate aminotransferase, *DM* diabetes mellitus, *irAEs* immune-related adverse events, *QTc* corrected QT

**Table 4:** Clinical information for irAE group

No.	irAE	CTCAE grade	Onset date	Nivolumab line	Duration of treatment with nivolumab	Treatment	Outcome
1	Diarrhea/colitis	1	60	5	228	Symptomatic therapy	Improved
	Appetite loss	2	158				
2	Pruritus	1	28	5	72	Observation	Improved
	Edema limbs	1	28			Symptomatic therapy	Improved
	QTc interval prolonged	1	42			Observation	Improved
3	Type 1 DM/Hyperglycemia	3	407	4	974	Insulin injection + DPP4 inhibitor	Improved
4	Pruritus	1	72	6	902	Observation	Improved
	Hypothyroidism	1	167			Thyroid hormone	Improved
	Type 1 DM/Hyperglycemia	3	195			Insulin injection	Improved
5	Dry skin	1	29	5	135	Observation	Improved
	Diarrhea/colitis	2	60			Corticosteroid	Improved
6	Diarrhea/colitis	3	93	5	350	Corticosteroid	Improved
7	Rash	1	11	4	266	Observation	Improved
8	Diarrhea/colitis	1	33	3	140	Observation	Improved
9	Peripheral motor neuropathy	3	3	5	107	Corticosteroid	Improved
10	Rash	2	13	4	118	Corticosteroid	Improved
	Diarrhea/colitis	1	13				
11	Adrenal insufficiency	3	143	6	109	Corticosteroid	Improved
12	Myalgia	2	16	3	194	Corticosteroid	Improved
13	AST increased	3	28	3	147	Observation	Improved
	ALT increased	2	28			Observation	Improved
14	Pneumonitis	3	32	4	63	Corticosteroid	Stable

*ALT* alanine aminotransferase, *AST* aspartate aminotransferase, *DM* diabetes mellitus, *DPP4* dipeptidyl peptidase, *irAEs* immune-related adverse events, *QTc* corrected QT

## Discussion

The toxicity profile of nivolumab in this study was similar to the ATTRACTION-2 study [12]. The AEs observed in the irAE group were manageable. There were no grade 4 or 5 adverse events related to nivolumab and no exacerbation of irAEs after detection. This study showed that irAEs were associated with efficacy of nivolumab in patients with AGC, as determined by favorable prognosis. In the irAE group, the ORR was 27.3% (95% CI, 9.8 to 56.6), the median PFS was 7.5 months (95% CI, 3.6 to 11.5), and the median OS was 16.8 months (95% CI, 4.4 to not reached). Judd J et al. reported the relation of irAEs with patient characteristics and outcomes in non-melanoma (head and neck squamous cell carcinoma, non-small cell lung cancer, renal cell carcinoma, and urothelial carcinoma) patients who received the PD-1 checkpoint inhibitors [27]; the ORR was 14% in patients with non-irAEs, 32% in patients with low-grade irAEs. Our results of a higher ORR in the irAE group were consistent with this previous report. Though it may not be appropriate to compare our data with those of non–small cell lung cancer and melanoma, a correlation between irAEs and tumor response in AGC patients who received nivolumab seems to be consistent among various types of cancers including AGC.

However, this type of analysis may have lead-time bias in that the short-term survivors may have a low risk of irAEs developing. The landmark analysis to minimize lead-time bias also proved the significant difference between irAE and non-irAE groups. Biagio R et al. reported 12- and 6-week landmark analysis in 195 patients with non-small cell lung cancer considering the lead-time bias due to the time-dependent onset of irAEs [28]. In their study, irAEs were significantly associated with improved clinical outcome in both the 12- and 6-week landmark analysis. In this study, 10 and 11 of 14 irAEs occurred within 2 and 3 months, respectively. Similarly, many irAEs were reported to be observed within 3 months in the ATTRACTION-2 trial [29]. From the point of treatment duration, the median PFS in ATTRACTION-2 study was 1.6 months (95% CI, 1.5 to 2.3); in our study, PFS as short as 1.4 months (95% CI, 1.2 to 1.6) in the non-irAE group. These results indicated that more than half of the patients discontinued nivolumab within 2 months. Therefore, it is considered reasonable to set the criteria of selecting patients by 2 or 3 months for the landmark analysis in this study. Additionally, the irAE group showed significantly longer OS and PFS than the non-irAE group in the landmark analysis, even after excluding the patients who had events within one, two and 3 months. This landmark analysis supports the hypothesis that the occurrence of irAEs is significantly associated with better outcomes of AGC patients.

Regarding the prognostic factors identified via multivariate analysis, number of metastatic sites ≥2, ALP high, and non-irAE group remained significantly associated with shorter OS in our study. More generally, a known prognostic index for AGC was developed based on the clinical trial, Japan Clinical Oncology Group (JCOG) 9912, which investigated superiority of irinotecan plus cisplatin and non-inferiority of oral S-1 compared with continuous infusion of 5-fluorouracil for patients with AGC [25]; this prognostic index consists of the following four independent risk factors for survival: performance status ≥1, number of metastatic sites ≥2, no prior gastrectomy, and elevated alkaline phosphatase (ALP). To analyze the impact of known prognostic factors, we adopted these four documented risk factors and occurrence of irAEs as covariates for multivariate analysis. We also performed the CIE method [26] and assessed the influence of other factors, such as age, sex, histologic type and HER2 status. Although we could analyze only a limited number of patient samples, it is speculated that occurrence of irAEs may be associated with survival even after adjusting other prognostic factors in AGC patients treated with nivolumab. Previous studies have reported that peripheral blood cell count or NLR in clinical course correlated with prognosis in several cancers [30]. However, in our study, it could not be said that these factors were useful biomarkers for predicting occurrence of irAE.

There were 14 patients who experienced irAEs in our study, and their irAEs were controlled after observation or treatment with immunosuppressive or endocrine therapies. Eight patients were able to continue nivolumab without treatment vacation. Four patients were able to be resume nivolumab treatment after temporary discontinuation. Two patients could not resume nivolumab treatment; due to disease progression in one patient and unrecovered nivolumab-related pneumonitis in the other. In general, management of irAEs in patients who receive immune checkpoint inhibitors has been recommended in the American Society of Clinical Oncology clinical practice guidelines [31]. In these guidelines, rechallenge of immune checkpoint inhibitors can be generally offered when symptoms and/or laboratory values revert to grade 1 or less, apart from some exceptional cases. Furthermore, it was reported that a subset of responders to PD-1 blockade present with a long-term clinical response even after discontinuation of the therapy [32]. Osa A et al. reported that prolonged nivolumab binding was detected more than 20 weeks after the last infusion, regardless of the total number of nivolumab infusions or type of subsequent treatment [33]. From this result, it can be proposed that we may resume immune checkpoint inhibitors after controlling irAEs. However, it should be taken into consideration that the management of irAEs should be performed adequately, and the restart of immune checkpoint inhibitor treatment should be decided safely under careful judgment.

This study has some limitations. First, the study is retrospective and conducted in a single center in Japan. Second, the sample size was small. Third, translational research to explore the mechanism and patient background of irAEs was not conducted. However, to the best of our knowledge, this is the first work to reveal an association between irAEs and efficacy of immune checkpoint inhibitors in AGC.

## Conclusions

Occurrence of irAEs was significantly associated with clinical outcomes of AGC patients treated with nivolumab. The mechanism of irAEs and patient background of those experiencing these events, which can be a biomarker of immune checkpoint inhibitors, should be clarified in the future.

## Data Availability

The datasets generated during the current study are not publicly available due to ethical restrictions, but are available from the corresponding author on reasonable request.

## Abbreviations

AGC: Advanced gastric cancer
ALP: Alkaline phosphatase
CI: Confidence interval
CIE: Change-in-estimate
ECOG PS: Eastern Cooperative Oncology Group Performance Status
HR: Hazard ratio
irAEs: Immune-related adverse events
MST: Median survival time
NLR: Neutrophil-to-lymphocyte ratio
ORR: Objective response rate
OS: Overall survival
PD-1: Programmed cell death-1
PFS: Progression-free survival
PR: Partial response
RECIST: Response evaluation criteria in solid tumors
SD: Stable disease
